# Expanding the Phenotypic Spectrum of APMR4 Syndrome Caused by a Novel Variant in *LSS* Gene

**DOI:** 10.1007/s12031-022-02074-y

**Published:** 2022-10-17

**Authors:** Nesma M. Elaraby, Hoda A. Ahmed, Neveen A. Ashaat, Sameh Tawfik, Mahmoud K. H. Ahmed, Nehal F. Hassib, Engy A. Ashaat

**Affiliations:** 1 Medical Molecular Genetics Department, Human Genetics and Genome Research Institute, National Research Centre, Cairo, Egypt; 2 Genetics Department, Ain Shams University, Cairo, Egypt; 3 Pediatric Department, Maadi Hospital, Cairo, Egypt; 4 Prenatal Diagnosis and Fetal Medicine Department, Human Genetics and Genome Research Institute, National Research Centre, Cairo, Egypt; 5 Orodental Genetics Department, Human Genetics and Genome Research Institute, National Research Centre, Cairo, Egypt; 6 Clinical Genetics Department, Human Genetics and Genome Research Institute, National Research Centre, Cairo, Egypt

**Keywords:** Whole-exome sequencing, Segregation, Alopecia-intellectual disability syndrome 4 (APMR4), LSS gene

## Abstract

Alopecia intellectual disability syndromes 4 (APMR4) is a very rare autosomal recessive condition caused by a mutation in the *LSS* gene present on chromosome 21. This syndrome has a clinical heterogeneity mainly exhibited with variable degrees of intellectual disability (ID) and congenital alopecia, as well. Eight families with 13 cases have been previously reported. Herein, we provide a report on an Egyptian family with two affected siblings and one affected fetus who was diagnosed prenatally. Whole-exome sequencing (WES) revealed a novel pathogenic missense variant (c.1609G > T; p.Val537Leu) in the lanosterol synthase gene (*LSS*) related to the examined patients. The detected variant was confirmed by Sanger sequencing. Segregation analyses confirmed that the parents were heterozygous. Our patient was presented with typical clinical manifestations of the disease in addition to new phenotypic features which included some dysmorphic facies as frontal bossing and bilateral large ears, as well as bilateral hyperextensibility of the fingers and wrist joints, short stature, umbilical hernia, and teeth mineralization defect. This study is the first study in Egypt and the 9th molecularly proven family to date. The aim is to expand the clinical and mutational spectrum of the syndrome. Moreover, the report gives a hint on the importance of prenatal testing and the proper genetic counseling to help the parents to take their own decision based on their beliefs.

## Introduction

Alopecia-intellectual syndromes (APMR) are extremely rare neuro-dermal disorders presented by ectodermal findings and neurological deficits. The cardinal signs of the syndromes are loss of scalp hair and eyelashes as well as intellectual disability (ID). APMR are four types classified based on the degree of ID, as well as associated clinical manifestations (Muzammal et al. 2021). Clinical heterogeneity was observed among types of APMR. Severe intellectual disability (ID) is found in relation to APMR3 (OMIM# 613,930) patients. Mild to moderate ID was noticed with APMR1 (OMIM# 203,650) and APMR2 (OMIM# 610,422). However, alopecia intellectual disability syndromes 4 (APMR4) (OMIM# 618,840) has a wider degree of affection when describing ID ranging from mild to severe (Besnard et al. 2019; Sailani Reza et al. 2017). APMR disorder predictive estimated prevalence is < 1:1,000,000 worldwide (Muzammal et al. 2021).

A few numbers of families with APMR have been reported worldwide (Muzammal et al. 2021)*.* The first affected family with APMR was described with alopecia and epilepsy in 1962 (Moynahan 1962). A new family with APMR exhibited new features in form of dental pyorrhea and teeth loss was diagnosed by Shokeir (1977). APMR4 having the wider intellectual disabilities clinical variability has been previously reported in eight families presented with 13 patients recruited from France, Switzerland, Belgium, and Doha in Qatar (Romano et al. 2018; Besnard et al. 2019).

Based on molecular characterization, four specific loci have been identified in which two APMR genes were described, and two other loci are still uncharacterized. The two identified genes were alpha 2-HS glycoprotein (*AHSG*) and lanosterol synthase (*LSS*) and mapped on chromosomes 3 and 21, respectively (Sailani Reza et al. 2017; Besnard et al. 2019). *LSS* gene is located on chromosome 21q22.3 and is composed of 23 exons and 732 amino acids. It encodes lanosterol synthase enzyme which converts (S)-2,3-oxidosqualene to lanosterol in the cholesterol biosynthesis pathway which has the key role of the metabolic pathway in the homeostasis of hair growth. Abnormal mutational expression of the *LSS* gene caused congenital cataracts (OMIM# 616,509) (Zhao et al. 2015), APMR4 (OMIM#618,840) (Romano et al. 2018; Besnard et al. 2019), and autosomal recessive hypotrichosis (OMIM# 618,275) (Romano et al. 2018).

Herein, a report on an Egyptian family with affected siblings presented with APMR4 and exhibited the classic neuro-dermal presentation in addition to newly described features. Prenatal genetic testing was carried out for the diagnosis of the mother’s 3rd pregnancy and revealed that the fetus also was affected. Our aim extends to reach proper genetic counseling concerning the affected fetus.

## Material and Methods

### Clinical Presentation

A 2-month girl was admitted to the ICU of a private hospital due to respiratory distress. Upon examination, the pediatrician thought that this girl could be syndromic. A specific geneticist was recalled for assessing the admitted patient. The girl suffered of complete hair loss of the scalp, eyelashes, and eyebrows. She had tendency to be microcephalic, and her head circumference was 36 (− 2.3 SD); however, weight and height were on mean for age and sex. A history was taken from the parents; they declared the presence of another elder sister with alopecia. Blood sample was taken from the admitted girl after signing an informed consent, and the parents were advised to seek professional genetic medical advice, diagnosis, and counseling.

**Fig. 1:**
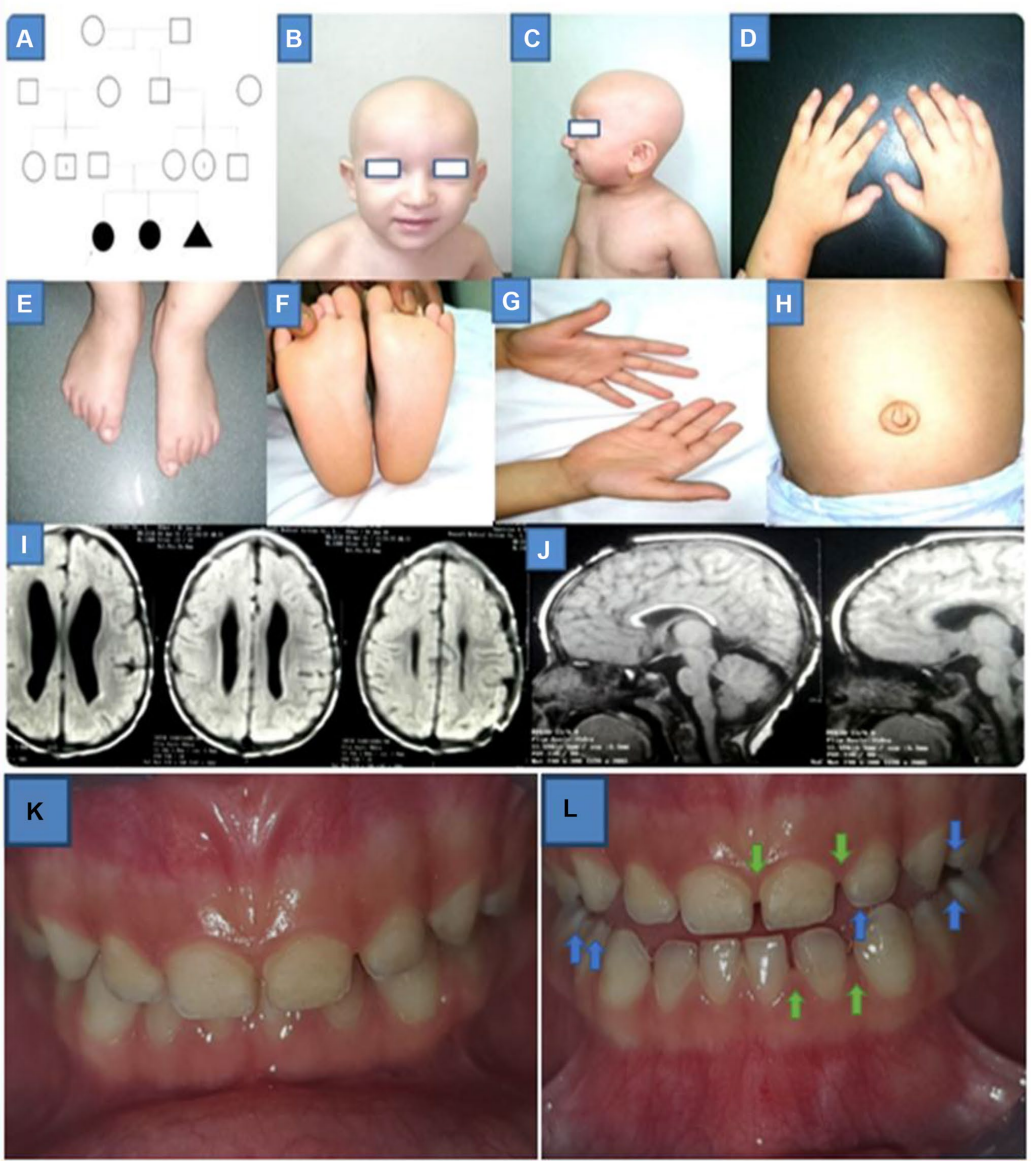
**A** Family pedigree. **B** and **C** Phenotypic manifestations of the proband. **D**, **E**, **F**, and **G** Shows no skin manifestations or nail dystrophy. **H** Umbilical hernia. **I** and **J** MRI findings. **K** Intraoral photo shows anterior deep overbite and normal intact healthy gingiva. **L** Enamel hypocalcification in upper anterior teeth, cusp tips of deciduous molars (blue arrows), and normal deciduous teeth spacing (green arrows)

Later, the family came to the Multiple Congenital Anomalies (MCAs) Clinic of the National Research Centre, Centre of Excellence, Cairo, Egypt. The girl child was 2 years and 6 months old of 1st-degree consanguineous parent. She was presented with congenital alopecia, the complete absence of eyelashes and eyebrows, and global developmental delay. Detailed family, medical, dental, and obstetric histories were taken from the parents. A family pedigree of up to three generations was drawn (Fig. 1A). The pregnancy and delivery histories were irrelevant. History of similarly affected female sib with congenital alopecia who died at 2 months due to respiratory distress was taken into consideration. Besides the cardinal signs of the syndrome, clinical examination of the affected proband showed new phenotypic findings which included some dysmorphic features presented with frontal bossing, and bilateral large ears (Fig. 1B, C). According to anthropometric measurements, growth retardation (weight was 8 kg, − 3.5 SD) and short stature (length was 77 cm, − 3.1 SD) were newly noticed phenotypes. Our affected girl had no signs of any skin manifestations with no nail dystrophy (Fig. 1D–G). Increasingly, an umbilical hernia and bilateral hyperextensibility of the fingers and wrist joints were also observed during the examination (Fig. 1H). The patient was microcephalic (head circumference was 43 cm, − 3.8 SD). Neurological examination revealed hypotonia with hyporeflexia. The genital examination was normal. Complete eye examination with fundus showed normal optic disk and macula with no refractive error. EEG was done and normal. MRI brain revealed moderate dilatation of the lateral ventricle with associated periventricular white matter dysmyelination and thin corpus callosum (Fig. 1I, J). IQ evaluation was done by using the Stanford Binet test and was 82 (low average scoring). Childhood Autism Rating Scale (CARS) was done with score of 27, which means that the proband does not have autistic features. Laboratory investigations for liver enzymes, thyroid, lipid profiles, and vitamin D were in the normal range. Extraoral examination exhibited short philtrum and thin upper lip. Orally, the examination revealed normal spacing of deciduous teeth with deep overbite as malocclusion (Fig. 1K). Enamel hypocalcification of upper anterior teeth and cusp tips of deciduous molars was noticed herein (Fig. 1L). No erythematous areas on the buccal mucosa were seen and the gingiva appeared very intact and healthy (Fig. 1L). The preliminary diagnosis went to neuro-dermal syndromes without reaching an accurate diagnosis. The whole-exome sequencing was the optimum choice to diagnose such a rare disorder. The study was approved by the Medical Research Ethics Committee of the National Research Centre (NRC), Cairo, Egypt, and the parents signed written informed consent. A blood sample was taken from the affected girl and the parents for molecular investigations.

### Molecular Analyses

#### Genomic DNA Extraction

Genomic DNA was extracted from peripheral blood samples of all participants using QIAamp DNA Mini Kit (Qiagen, Hilden, Germany). DNA concentration was determined using Qubit™ Fluorometer (Thermo Fisher Scientific, Inc.). After that, DNA samples were subjected to electrophoresis in 1% agarose gel and the optical density ratio was used to confirm its integrity and purity.

#### Whole-Exome Sequencing

To find disease-causing mutation, WES was performed on the DNA sample of the affected patients. Library preparation and a sequencing run were performed using the Illumina HiSeq sequencer (Illumina, USA). Raw sequencing reads were aligned to the human hg19 reference genome using Burrows-Wheeler Aligner (Li and Durbin 2010). Local realignment, variant calling, and SNP/Indel analyses were carried out following Genome Analysis Tool Kit (GATK) (DePristo et al. 2011; Van der Auwera et al. 2013), Annovar (Wang et al. 2010), and SnpSift (Cingolani et al. 2012) software.

#### Variant Segregation

Sanger sequencing was performed to confirm that prioritized variant segregated consistently among parents and available family members according to the predicted mode of inheritance. We designed primers targeting *LSS* exon which harbor the filtered variant of interest using primer3 software (http://bioinfo.ut.ee/primer3-0.4.0/) (Untergasser et al. 2012). The amplified DNA fragments were purified and sequenced in both directions using ABI Prism 3500 Genetic Analyzer (Applied Biosystems, Waltham, MA, USA). Variants were named based on Human Genome Variation Society nomenclature recommendations (Den Dunnen et al. 2016). The standards of the American College of Medical Genetics and Genomics (ACMG) were used to classify the level of variant pathogenicity, i.e., pathogenic, likely pathogenic, variant of unknown significance (VUS), benign, or likely benign (Richards et al. 2015).

#### In-Silico Functional/Structural Prediction Tools

Functional predictions of *LSS* detected variant were determined from sequence homology-based algorithms including Sorting Intolerant From Tolerant (SIFT; http://sift.bii.a-star.edu.sg/), PolyPhen-2Polymorphism phenotyping 2.0 (http://genetics.bwh.harvard.edu/pph2/), PROVEAN (http://provean.jcvi.org/index.php), mutation tester (https://www.mutationtaster.org/), CADD (Combined Annotation Dependent Depletion; https://cadd.gs.washington.edu/), M-CAP (http://bejerano.stanford.edu/mcap/), List-S2 (https://list-s2.msl.ubc.ca/), and MutPred (http://mutpred.mutdb.org/).

Different computational protein stability tools were used to predict the effects of the detected variant on the stability of *LSS* protein, PREMPS (https://lilab.jysw.suda.edu.cn/research/PremPS/), I-Mutant2.0 (https://folding.biofold.org/cgi-bin/imutant2.0.cgi), MUpro (http://mupro.proteomics.ics.uci.edu/), and DynaMut 2 (https://biosig.unimelb.edu.au/dynamut/) tools. The outputs from these tools predicted the change in Gibbs free energy (DDG or ΔΔ*G*) values via measuring the change in energy between the folded and unfolded states (or between wild-type and the variant protein). It should be noted that each predicted tool has a definite score and threshold to evaluate the mutation to be either stabilizing or destabilizing to the protein structure.

The protein secondary structure of *LSS* was visualized and annotated by using the online SOPMA server (https://npsa-prabi.ibcp.fr/cgibin/npsa automat.pl) to predict the effect of the predicted variant on domain structure, which explained the distributions of beta sheet, alpha helix, and coil. The human *LSS* amino acid sequence was obtained in FASTA format from UniProt database (http://www.uniprot.org/). The three-dimensional (3D) structure of *LSS* was downloaded from the Protein Data Bank (PDB) and attribution of the residue position to the protein function for wild and mutant sequence was analyzed by using SWISS-MODEL template library (http://swissmodel.expasy) that was predicted using the online Swissmodel Webserver.

## Results

**Fig. 2:**
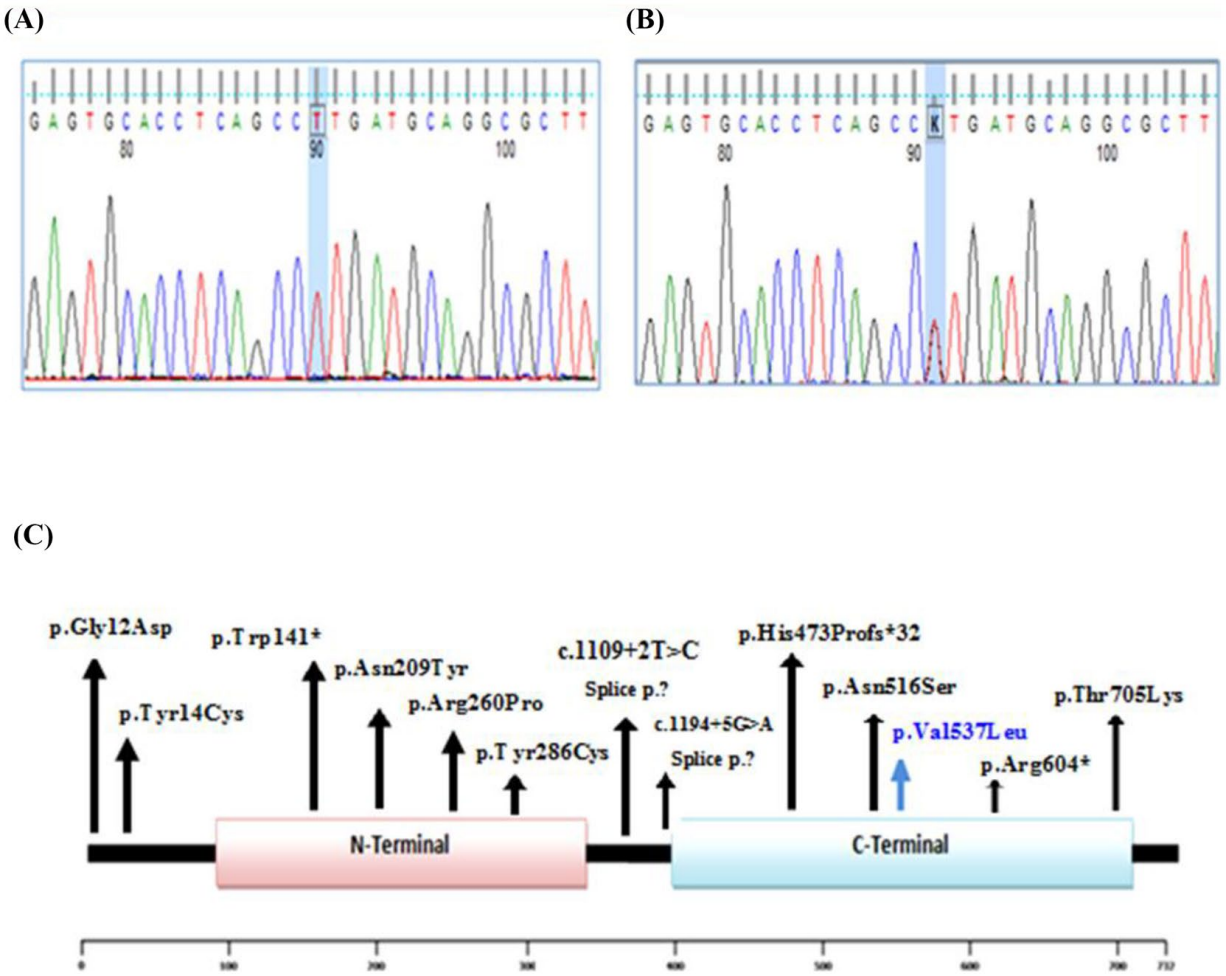
Segregation analysis and localization of the detected variant in LSS protein. **A** and **B** Sanger sequencing chromatographs of the homozygous variant c.1609G > T detected in the affected female and the heterozygous carrier state of the parents. **C** The schematic of LSS protein structure and the positions of the novel p.Val537Leu mutation (blue) and those previously reported in the *LSS* gene (black) causing APMR4 syndrome

After variant filtering, WES analysis of the affected patients revealed a novel homozygous missense variant c.1609G > T found in exon 17 of *LSS* gene at C terminal domain (NM_002340.6, NP_002331.3). This variant results from a valine-to-leucine substitution at position 537 (p.Val537Leu) (Fig. 2A–C). Sanger sequencing analysis was carried out to confirm the finding (c.1609G > T [p.Val537Leu]) and to investigate familial segregation of the detected variant. Homozygous missense variant was detected in the affected patients (Fig. 2A) and the parents were heterozygous carriers (Fig. 2B). The p.Val537Leu variant was predicted to be likely pathogenic according to the American College of Medical Genetics and Genomics (ACMG) guidelines based on the evidence chain (PM2, PP2, PP3). Furthermore, the variant was not found in any of the large population databases (dbSNP, 1000G, and gnomAD) or in our in-house database of 55 Egyptian exomes.

**Fig. 3:**
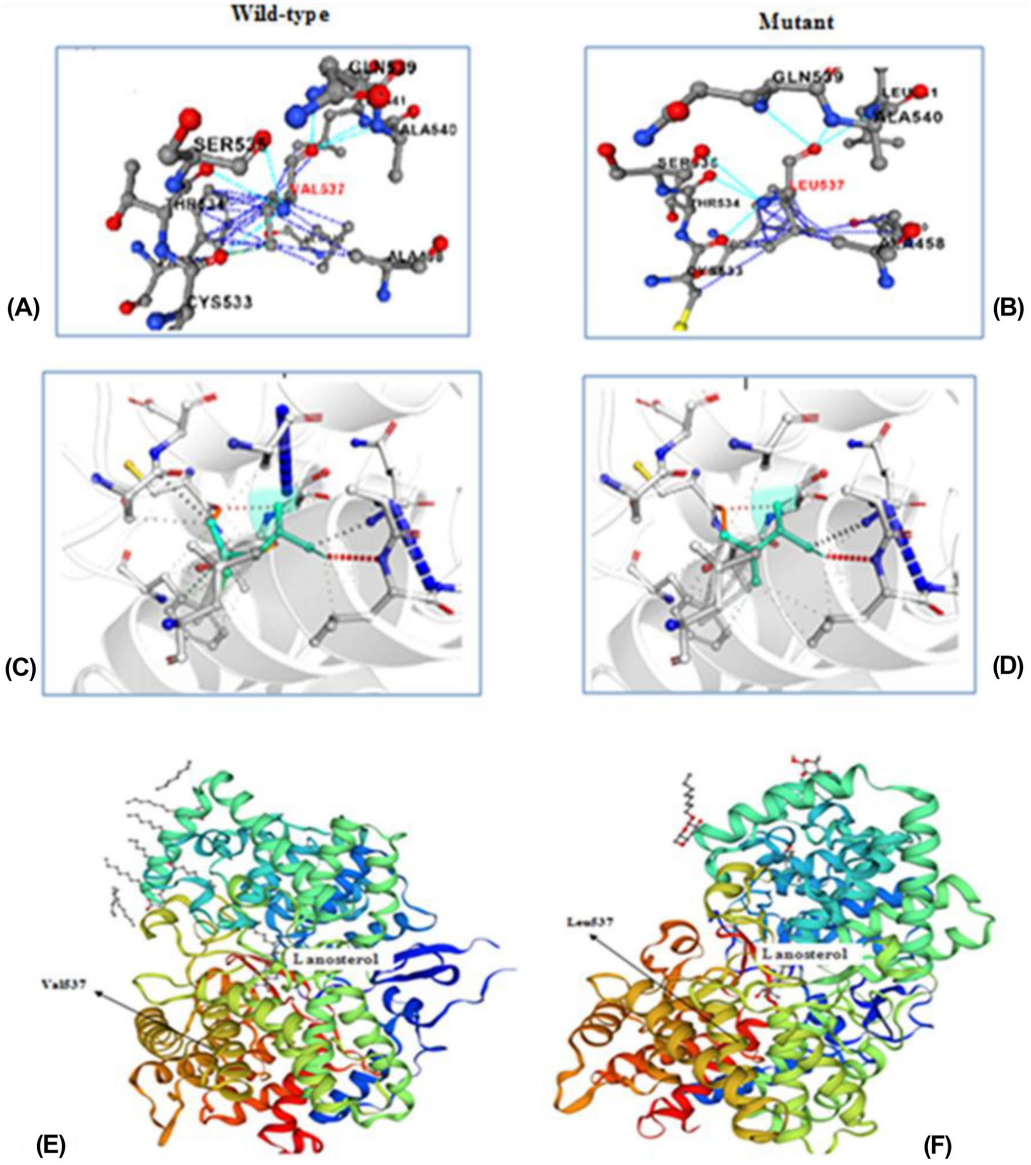
Interaction prediction analysis for p.Val537Leu using different in-silico tools. **A** and **B** The interatomic interaction of V537 with the surrounding residue in WT and of L537 in V537L mutant using Premps tool. **C** and **D** Interactions in WT and mutant residue by using DynaMut tool are colored in light-green and are also represented as sticks alongside with the surrounding residues which are involved on any type of interactions, whereas V and L represent the valine to leucine substitution at 537 position. **E** and **F** SWISS-MODEL predicted 3D for WT and mutant structures based on the X-ray structure (PDB code: 1W6K) X-ray diffraction, 2.10 Å, which contains 9 ligands: 8 × octyl beta-D-glucopyranoside and 1 × lanosterol

To obtain more confidence results for the pathogenicity of p.Val537Leu variant, different in-silico prediction tools were utilized and were predicted to be probably damaging, damaging, and deleterious whereas just one algorithm (PROVEAN) was predicted to be “neutral.” The prediction algorithms used in this study are given in Table 1. Additionally, the impact of this variant on protein thermodynamic stability was determined by measuring the changes in the Gibbs free energy of unfolding (∆∆*G*) between wild-type (WT) and Mutant protein. Based on the results obtained from Premps, I-Mutant 2.0, and DynaMut 2 tools, ΔΔ*G* values showed that the detected variant was reducing protein activity by decreasing its protein stability with ΔΔ*G* values (= 0.78, 0.3, and 0.52 kcal/mol, respectively), and the results of MUpro tool predicted this variant to be deleterious as it caused a decrease in the protein stability with ∆∆*G* =  − 0.635 kcal/mol. In addition, from Premps results this variant was found in the core of the protein. The protein stability tool findings are described in Fig. 3A–D.

**Table 1:** In-silico variant effect prediction tools and ACMG classification of *LSS* gene variant

**Gene**	**Mutation**	**Polyphen**	**M-CAP**	**MutationTaster**	**PROVEAN**	**LIST-S2**	**SIFT**	**CADD score**	MUPro	MutPred	ACMG classification
*LSS* (NM_002340.6)	c.1609G > T p.Val537Leu	Probably damaging (score of 0.97)	Damaging (score: 0.1316)	Deleterious	Neutral	Damaging (score: 0.98)	Damaging (0.02)	24.9 Deleterious	∆∆*G* = − 0.635 (decrease stability)	Pathogenic (score: 0.624)	Likely pathogenic (PM2,PP2, PP3)

Polyphen score 0.97 indicates that the variant is predicted to be probably damaging based on a probabilistic score (0.0–0.15 benign, 0.15–0.85 possibly damaging, and 0.85–1.0 probably damaging). M-CAP score is presented between 0 and 1. PROVEAN threshold index is − 2.5. If the final score of an amino acid change is below the cutoff, it is predicted to be “deleterious”; otherwise, it is classified as “neutral.” SIFT score: ≤ 0.05 is predicted to be deleterious; otherwise, ≥ 0.05 it is considered to be tolerated. In CAAD, C-score of ≥ 10 indicates that variants are predicted to be the 10%, a score of ≥ 20 indicates the 1% most deleterious, and so on. MutPred: > the 0.5 threshold is interpreted as a pathogenic amino acid substitution. MUPro score < 0 means the mutation decreases the protein’s stability; conversely, a score > 0 means the mutation increases protein stability

*M-CAP* Mendelian Clinically Applicable Pathogenicity, *PROVEAN* Protein Variation Effect Analyze, *CADD* Combined Annotation Dependent Depletion, *SIFT* Sort Intolerant from Tolerant, *ACMG* American College of Medical Genetics and Genomics

Our findings show that the detected variant in the *LSS* protein at p.Val537Leu induce pathogenicity. The human *LSS* amino acid sequence was obtained in FASTA format from UniProt ID: P48449. The structural models of WT and mutant *LSS* monomeric proteins were based on the X-ray structure (PDB code: 1W6K) and were predicted by SWISS-MODEL method as given in Fig. 3E, F. This template 1W6K served as the foundation for our model backbone and structure prediction.

**Fig. 4:**
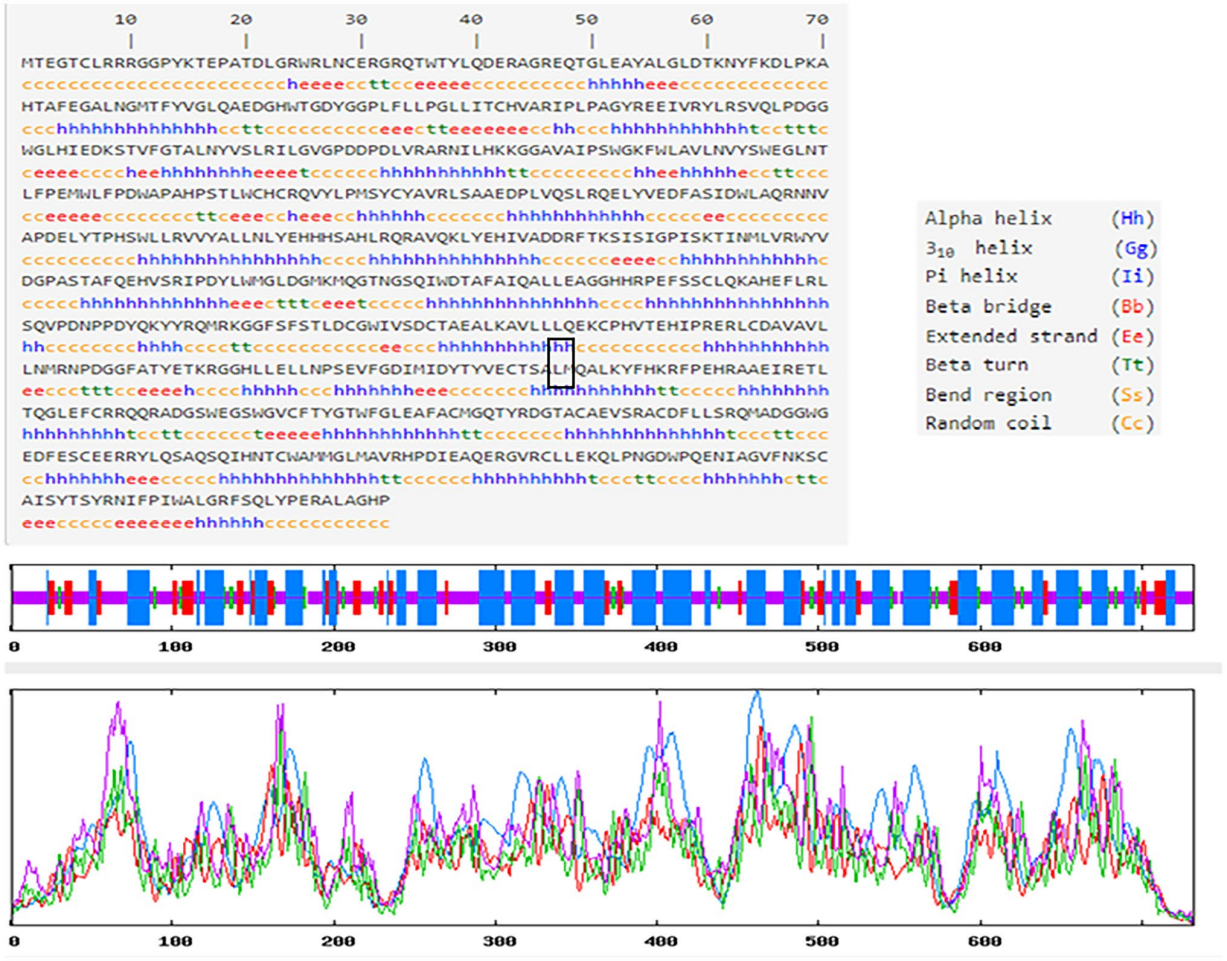
Secondary structure model predicted for mutant sequence by the SOPMA server, showing the secondary structural model components: alpha helix, beta turn, extended strands, and random coil. The small rectangular box shows the mutant residue

Subsequently, the impact of amino acid alteration on domain structure was evaluated by comparing wild-type secondary structure and mutant sequences. The results indicated the mutated structure was predicted to consist of many alpha helixes that are 317 (43.31%), followed by 294 random coils (40.16%), 87 extended strands (11.89%), and 44 beta turns (6.01%) compared to 308 (42.08%), followed by 296 (40.44%), 85 (11.61%), and 43 (5.87%), respectively, for WT secondary structure (Fig. 4).

### Prenatal Genetic Testing

The mother got pregnant for the third time and prenatal testing was performed at the first trimester of pregnancy (11^+0^ till 13^+6^ weeks of gestation). Twenty to thirty milliliters of amniotic fluid was gently aspirated into syringes, transferred to sterile tubes, and transported at room temperature to the laboratory for processing the amniotic fluid sample. The amniotic fluid DNA was extracted using the QIAamp DNA Mini Kit (Qiagen, Germany) according to the manufacturer’s protocol; then, we applied Sanger sequencing technique for targeted mutation detected. Results of the prenatal testing revealed that the fetus was similarly affected. Genetic counseling was offered to the parents to help them to achieve their proper decision based on accurate diagnosis, prenatal testing, and their own values and beliefs. The mother at the end did an elective abortion after the prenatal testing.

## Discussion

Cholesterol synthesis is a complex cellular process that requires a lanosterol synthase (LSS) enzyme which is the rate-limiting enzyme in cholesterol synthesis pathway. Cholesterol is important for the structure of central nervous system and hair follicle morphology. Deficiency in this enzyme is associated with skin lesions, cataracts, and congenital anomalies (Chen and Liu 2017; Romano et al. 2018; Besnard et al. 2019).

Whole-exome sequencing revealed a novel missense variant (c.1609G > T; p.Val537Leu) in the studied family. This variant is predicted to be deleterious by different in-silico tools (Table 1). We confirmed the segregation of this variant with Sanger sequencing and showed that the variant was “heterozygous” in the parents. Our patient exhibited congenital alopecia, microcephaly, global developmental delay, and hypotonia with hyporeflexia. Our classical clinical results are in accordance with two previous studies conducted by Romano et al. (2018) and Besnard et al. (2019).

 The newly found features including dysmorphic features as frontal bossing and bilateral large ears, growth retardation, short stature, umbilical hernia, joint hyperextensibility, and teeth mineralization defect have not been reported previously. Besnard et al. (2019) observed interfamilial and intrafamilial phenotypic variability among families affected with APMR; different *LSS* variants between families explain the interfamilial phenotypic variability, but the etiology of the intrafamilial variability remains unclear. Comparisons between previously reported patients and our patient were summarized in Table 2.

**Table 2:** Summary of clinical, laboratory, and molecular findings in APMR 4 patients to data

	**Family 1**	**Family 2**	**Family 3**	**Family 4**	**Family 5**	**Family 6**	**Family 7**	**Family 8**	**Our family (Family 9)**
1.**Clinical findings**									
** No of patients**	2	2	2	2	2	1	1	1	3
** Consanguinity**	X	x	√	√	√	√	x	√	√
** Congenital cataracts**	X	x	X	x	x	x	x	x	x
** AR hypotrichosis (congenital alopecia)**	√	√	√	√	√	√	√	√	√
** Ectodermal phenotypes**	X	x	√	x	√	x	x	x	x
** Developmental delay**	√	√	√	√	√	√	√	√	√
** Facial dysmorphism**	X	x	x	x	x	x	x	x	√
** Short stature**	X	x	x	x	x	x	x	x	√
** Growth retardation**	X	x	x	x	x	x	x	x	√
** Umbilical hernia**	X	x	x	x	x	x	x	x	√
** Hyperextensibility of joints**	X	x	x	x	x	x	x	x	√
** Tooth mineralization defect**	X	x	x	x	x	x	x	x	√
** Dental deep overbite**	X	x	x	x	x	x	x	x	√
** Total cholesterol**	Normal	Normal	ND	Low Normal	ND	Normal	ND	Normal	Normal
2.**Molecular findings**									
** Nucleotide change**	c.625A > T c.423G > A	c.1547A > G c.2114C > A	c.779G > C c.1194 + 5G > A	c.1109 + 2 T > C	c.857A > G c.1810C > T	c.1417dup c.41A > G	c.35G > A	c.1955C > T	c.1609G > T
** Amino acid change (AA)**	p.Asn209Tyr p.Trp141*	p.Asn516Ser p.Thr705Lys	p.Arg260Pro Splice p.?	Splice p.?	p.Tyr286Cys p.Arg604*	p.His473Profs*32 p.Tyr14Cys	p.Gly12Asp	p.Thr652Ile	p.Val537Leu
** Zygosity**	Comp. het	Comp. het	Comp. het	Homo	Comp. het	Comp. het	Homo	Homo	Homo
** Location**	N-term	C-term	N-term toward C-term	Toward N-term	N-term C-term	C-term N-term	N-term	C-term	C-term
** References**	Romano et al. (2018)	Besnard et al. (2019)							**This study**

*AR* autosomal recessive, *ND* not determined, *Homo* homozygous, *Comp. het.* compound heterozygous, C*-term* C terminal, *N-term* N terminal

Zhao et al. (2015) reported two families with congenital cataract caused by *LSS* variants. However, our patient had a normal eye examination. Chen and Liu (2017) reported another family with an affected child having congenital alopecia and micropenis. On the other hand, normal genitalia was present in the examined girl case, herein. The discrepancy in the results reported by Chen and Liu (2017) regarding small penis is mainly related to gender characterization. Gender could be taken into consideration during the examination of APMR4 syndrome to spot genitalia abnormalities.

Recently, Romano et al. (2018) reported three unrelated families with *LSS* mutations associated with hypotrichosis simplex but intellectual disability was observed in siblings from one of these families. Clinical variability was also observed in other syndromes associated with cholesterol biosynthesis deficiency such as Smith–Lemli–Opitz syndrome (Nowaczyk and Irons 2012). Some LSS mutations cause alopecia alone and others cause alopecia with intellectual disability; this clinical heterogeneity remains unclear. The degree of normal function protein may be related to this clinical variability. Romano et al. (2018) reported that mutation in the C terminal domain is associated with ocular manifestations while mutations in the N terminal domain are associated with hair loss. Those findings are in contrast with our patient who had a mutation in the C terminal domain and presented with alopecia with no ocular manifestations.

Lanosterol synthase gene is one of the proteins which have an indirect role in the metabolism of vitamin D, mineral deposition, and osteogenic differentiation (Takahashi et al. 2013). Through complicated procedures, the squalene chain controlled by squalene monooxygenase enzyme regulates the cholesterogenesis via catalysis process by the lanosterol synthase gene which is a precursor of cholesterol biosynthesis (Pinto and Cooper 2014). The lipid cholesterol enters in the metabolism of vitamin D, specifically vitamin D3 (Idoko et al. 2020). Vitamin D has a crucial impact on tooth mineralization; its deficiency leads to rachitic teeth (Swapna and Abdulsalam 2021). Our patient presented with a teeth mineralization defect. It could be justified that the mutation in the *LSS* gene affects cholesterol and vitamin D metabolism, consequently teeth mineralization.

In conclusion, this is the first study in Egypt that describes APMR4 syndrome, and the ninth molecularly proven family reported to date and expanding the clinical and the mutational spectrum of the syndrome. Our results emphasize the importance of genetic testing of patients with APMR, in order to provide prenatal diagnosis and proper genetic counseling for families with this disorder.

## Data Availability

The data supporting the findings of this study are available from the corresponding author upon request.

## References

[CR1] Besnard T, Sloboda N, Goldenberg A, Küry S, Cogné B, Breheret F, Isidor B (2019). Biallelic pathogenic variants in the lanosterol synthase gene LSS involved in the cholesterol biosynthesis cause alopecia with intellectual disability, a rare recessive neuroectodermal syndrome. Genet Med.

[CR2] Chen X, Liu L (2017). Congenital cataract with *LSS* gene mutations: a new case report. J Pediatr Endocrinol Metab.

[CR3] Cingolani P, Patel VM, Coon M et al (2012) Using *Drosophila melanogaster* as a model for genotoxic chemical mutational studies with a new program. SnpSift Front Genet 3(35). 10.3389/fgene.2012.00035. eCollection 2012 10.3389/fgene.2012.00035 PMC3304048 22435069

[CR4] Den Dunnen JT, Dalgleish R, Maglott DR, Hart RK, Greenblatt MS, McGowan-Jordan J, Roux A, Smith T, Antonarakis SE, Taschner PEM (2016). HGVS recommendations for the description of sequence variants: 2016 update. Hum Mutat.

[CR5] DePristo MA, Banks E, Poplin R (2011). A framework for variation discovery and genotyping using next-generation DNA sequencing data. Nat Genet.

[CR6] Idoko A, Ugwudike PO, Ayomide TA, Blessing NO (2020). Cholesterol and its implications — a review. Univ J Pharm Res.

[CR7] Li H, Durbin R (2010). Fast and accurate long-read alignment with Burrows-Wheeler transform. Bioinformatics.

[CR8] Moynahan EJ (1962). Familial congenital alopecia, epilepsy, mental retardation with unusual electroencephalograms. Proc R Soc Med.

[CR9] Muzammal M, Ahmad S, Ali MZ, Khan MA (2021). Alopecia-mental retardation syndrome: molecular genetics of a rare neuro-dermal disorder. Ann Hum Genet.

[CR10] Nowaczyk MJM, Irons MB (2012). Smith–Lemli–Opitz syndrome: phenotype, natural history, and epidemiology. Am J Med Genet C Semin Med Genet.

[CR11] Pinto JT, Cooper AJ (2014). From cholesterogenesis to steroidogenesis: role of riboflavin and flavoenzymes in the biosynthesis of vitamin D. Adv Nutr 1.

[CR12] Richards S, Aziz N, Bale S, Bick D, Das S, Gastier-Foster J, Grody WW, Hegde M, Lyon E, Spector E (2015). Standards and guidelines for the interpretation of sequence variants: a joint consensus recommendation of the American College of Medical Genetics and Genomics and the Association for Molecular Pathology. Genet Med.

[CR13] Romano M, Tafazzoli A, Mattern M (2018). Bi-allelic mutations in LSS, encoding lanosterol synthase, cause autosomal-recessive hypotrichosis simplex. Am J Hum Genet.

[CR14] Sailani Reza M, Jahanbani F, Nasiri J (2017). Association of AHSG with alopecia and intellectual disabilities (APMR) syndrome. Hum Genet.

[CR15] Shokeir MH (1977). Universal permanent alopecia, psychomotor epilepsy, pyorrhea and mental subnormality. Clin Genet.

[CR16] Swapna LA, Abdulsalam R (2021). Vitamin D deficiency and its effects on tooth structure and pulpal changes. Open Access Maced J Med Sci.

[CR17] Takahashi K, Ogura N, Aonuma H, Ito K, Ishigami D, Kamino Y, Kondoh T (2013). Bone morphogenetic protein 6 stimulates mineralization in human dental follicle cells without dexamethasone. Arch Oral Biol.

[CR18] Untergasser A, Cutcutache I, Koressaar T, Ye J, Faircloth BC, Remm M, Rozen SG (2012). Primer3—new capabilities and interfaces. Nucleic Acids Res.

[CR19] Van der Auwera, GA, Carneiro MO, Hartl C et al (2013) From FastQ data to high-confidence variant calls: the Genome Analysis Toolkit best practices pipeline. Curr Protoc Bioinforma 43(11.10.1–33). 10.1002/0471250953.bi1110s43 10.1002/0471250953.bi1110s43 PMC4243306 25431634

[CR20] Wang K, Li M, Hakonarson H (2010). ANNOVAR: functional annotation of genetic variants from high-throughput sequencing data. Nucleic Acids Res.

[CR21] Zhao L, Chen XJ, Zhu J (2015). Lanosterol reverses protein aggregation in cataracts. Nature.

