# Correction to: Expanding the Phenotypic Spectrum of APMR4 Syndrome Caused by a Novel Variant in *LSS* Gene

**DOI:** 10.1007/s12031-024-02233-3

**Published:** 2024-06-01

**Authors:** Nesma M. Elaraby, Hoda A. Ahmed, Neveen A. Ashaat, Sameh Tawfik, Mahmoud K. H. Ahmed, Nehal F. Hassib, Engy A. Ashaat

**Affiliations:** 1 Medical Molecular Genetics Department, Human Genetics and Genome Research Institute, National Research Centre, Cairo, Egypt; 2 Genetics Department, Ain Shams University, Cairo, Egypt; 3 Pediatric Department, Maadi Hospital, Cairo, Egypt; 4 Prenatal Diagnosis and Fetal Medicine Department, Human Genetics and Genome Research Institute, National Research Centre, Cairo, Egypt; 5 Orodental Genetics Department, Human Genetics and Genome Research Institute, National Research Centre, Cairo, Egypt; 6 Clinical Genetics Department, Human Genetics and Genome Research Institute, National Research Centre, Cairo, Egypt

**Correction to: Journal of Molecular Neuroscience (2022) 72:2242-2251** **https://doi.org/10.1007/s12031-022-02074-y**

The paper "Expanding the Phenotypic Spectrum of APMR4 Syndrome Caused by a Novel Variant in LSS Gene and Review of Literature" presenting a rare case. The promotion committee objects to the review of literature in the title. This correction is performed to remove "and Review of Literature" from the title as it is necessary for all authors.

The original article has been corrected.

